# CXCL10 Is an Agonist of the CC Family Chemokine Scavenger Receptor ACKR2/D6

**DOI:** 10.3390/cancers13051054

**Published:** 2021-03-02

**Authors:** Andy Chevigné, Bassam Janji, Max Meyrath, Nathan Reynders, Giulia D’Uonnolo, Tomasz Uchański, Malina Xiao, Guy Berchem, Markus Ollert, Yong-Jun Kwon, Muhammad Zaeem Noman, Martyna Szpakowska

**Affiliations:** 1 Department of Infection and Immunity, Immuno-Pharmacology and Interactomics, Luxembourg Institute of Health (LIH), L-4354 Esch-sur-Alzette, Luxembourg; andy.chevigne@lih.lu (A.C.); max.meyrath@lih.lu (M.M.); nathan.reynders@lih.lu (N.R.); giulia.duonnolo@lih.lu (G.D.); tomasz.uchanski@lih.lu (T.U.); markus.ollert@lih.lu (M.O.); 2 Department of Oncology, Tumor Immunotherapy and Microenvironment (TIME), Luxembourg Institute of Health (LIH), L-1526 Luxembourg City, Luxembourg; bassam.janji@lih.lu (B.J.); malina.xiao@lih.lu (M.X.); berchem.guy@chl.lu (G.B.); muhammadzaeem.noman@lih.lu (M.Z.N.); 3 Faculty of Science, Technology and Communication, University of Luxembourg, L-4365 Esch-sur-Alzette, Luxembourg; 4 Centre Hospitalier du Luxembourg, Department of Hemato-Oncology, L-1210 Luxembourg City, Luxembourg; 5 Department of Dermatology and Allergy Center, Odense Research Center for Anaphylaxis, University of Southern Denmark, DK-5000 Odense C, Denmark; 6 Disease Modeling and Screening Platform (DMSP), Luxembourg Institute of Health (LIH), L-1445 Strassen, Luxembourg; yong-jun.kwon@lih.lu

**Keywords:** D6, ACKR2, CXCL10, IP-10, scavenger, ACKR3, CXCR3, CXCL12, CXCL2, CD26, DPP4, NanoBiT, NanoBRET

## Abstract

**Simple Summary:**

The atypical chemokine receptor ACKR2 plays an important role in the tumour microenvironment. It has long been considered as a scavenger of inflammatory chemokines exclusively from the CC family. In this study, we identified the CXC chemokine CXCL10 as a new strong agonist ligand for ACKR2. CXCL10 is known to drive the infiltration of immune cells into the tumour bed and was previously reported to bind to CXCR3 only. We demonstrated that ACKR2 acts as a scavenger reducing the availability of CXCL10 for CXCR3. Our study sheds new light on the complexity of the chemokine network and the potential role of CXCL10 regulation by ACKR2 in tumour immunology.

**Abstract:**

Atypical chemokine receptors (ACKRs) are important regulators of chemokine functions. Among them, the atypical chemokine receptor ACKR2 (also known as D6) has long been considered as a scavenger of inflammatory chemokines exclusively from the CC family. In this study, by using highly sensitive β-arrestin recruitment assays based on NanoBiT and NanoBRET technologies, we identified the inflammatory CXC chemokine CXCL10 as a new strong agonist ligand for ACKR2. CXCL10 is known to play an important role in the infiltration of immune cells into the tumour bed and was previously reported to bind to CXCR3 only. We demonstrated that ACKR2 is able to internalize and reduce the availability of CXCL10 in the extracellular space. Moreover, we found that, in contrast to CC chemokines, CXCL10 activity towards ACKR2 was drastically reduced by the dipeptidyl peptidase 4 (DPP4 or CD26) N-terminal processing, pointing to a different receptor binding pocket occupancy by CC and CXC chemokines. Overall, our study sheds new light on the complexity of the chemokine network and the potential role of CXCL10 regulation by ACKR2 in many physiological and pathological processes, including tumour immunology. Our data also testify that systematic reassessment of chemokine-receptor pairing is critically needed as important interactions may remain unexplored.

## 1. Introduction

Chemokines are small (8–14 kDa) soluble cytokines that guide directional cell migration and orchestrate many important processes, including leukocyte recruitment during immunosurveillance. They are also involved in numerous inflammatory diseases and the development and spread of many cancers. Based on the presence of specific cysteine motifs in their N termini, chemokines are divided into four classes: CC, CXC, XC and CX3C. Their receptors belong to the G protein-coupled receptor (GPCR) family and are accordingly classified as CCR, CXCR, XCR and CX3CR, depending on the chemokine class they bind. Over the past years, a subfamily of four chemokine receptors has emerged as important regulators of chemokine functions. These receptors are termed atypical chemokine receptors (ACKR1-4) due to their inability to trigger a G protein-dependent signalling or directly induce cell migration in response to chemokine binding [1,2]. Nevertheless, ACKRs do play an important role within the chemokine-receptor network by shaping the gradient of chemokines, thereby regulating their effect on cells expressing their respective classical chemokine receptors. Most ACKRs have the ability to constitutively cycle between the cell membrane and the intracellular compartments, internalizing and directing for degradation the chemokines that they bind [1,3,4,5]. Although this activity was previously considered to mainly rely on β-arrestins, recent studies showed that alternative mechanisms can drive chemokine scavenging by ACKRs [6,7,8,9,10,11].

ACKR2 (formerly D6 or CCBP2) has been long reported to bind inflammatory chemokines exclusively from the CC family. ACKR2 main ligands include CCL2-8, CCL11-13, CCL17 and CCL22, which are agonists of the classical receptors CCR1-5 [12,13,14,15]. By scavenging this large spectrum of inflammatory chemokines, ACKR2 drives the resolution phase of inflammation and prevents exacerbated immune responses [16,17,18,19,20,21]. ACKR2 is expressed on lymphatic endothelial cells, epithelial cells, trophoblasts in placenta and some subsets of leukocytes, including alveolar macrophages and innate-like B cells [22,23,24]. Owing to its anti-inflammatory effect, ACKR2-deficient mice show an increased number of circulating inflammatory monocytes [25] and neutrophils [26,27], as well as defects in lymphatic vessel density and function [28]. ACKR2 was also shown as an important regulator of chemokines in inflammatory and autoimmune diseases, notably in psoriasis [18,29,30,31]. A scavenging-independent activity of ACKR2 has also been reported in apoptotic neutrophils, where ACKR2 was proposed to present chemokines to macrophages and promote inflammation resolution by shifting their phenotype [32,33].

Importantly, ACKR2 plays diverse and complex roles in tumour biology from initiation to metastasis [27,34,35]. ACKR2-deficient mice were shown to be more prone to tumour development but display increased tumour natural killer (NK) cell infiltration and circulating neutrophils, while opposing effects were reported regarding ACKR2 involvement in tumour dissemination [27,34,36]. Besides CC inflammatory chemokines, several CXC chemokines play important roles in inflammatory responses and are also found as part of tumour-associated inflammatory signatures [37,38]. In particular, the interferon gamma-induced chemokine CXCL10, also known as IP-10, reported to sustain tumour growth via autocrine loops [39] and to drive T lymphocytes and NK cells through activation of CXCR3 [37,40,41], is often upregulated in the same manner or simultaneously with CC inflammatory chemokines [42].

In this study, by applying highly sensitive assays monitoring β-arrestin recruitment, we identified CXCL10, previously known to exclusively bind to CXCR3, as a high-affinity agonist for ACKR2. This finding expands the panel of ACKR2 ligands to the CXC chemokine family and at the same time highlights the need for a systematic reassessment of chemokine-receptor pairing, as important interactions may remain unexplored.

## 2. Materials and Methods

### 2.1. Cells and Proteins

HEK-ACKR2 cell line stably expressing human or mouse ACKR2 were established by transfection of HEK293T cells (ATCC, Manassas, VA, USA) with pIRES-puro vector (Addgene, Watertown, MA, USA ) encoding the human or mouse ACKR2 and subsequent puromycin selection (5 μg/mL). Receptor surface expression was verified by flow cytometry using hACKR2-specific mAb (clone 196124, R&D Systems, Minneapolis, MI, USA) or polyclonal mACKR2-specific antibody (ab1656, Abcam, Cambridge, UK). The absence of CXCR3 at the cell surface was confirmed using mAb clone 1C6 and the corresponding isotype control (BioLegend, San Diego, CA, USA). The B16.F10 and U87.MG cell lines were purchased from ATCC. Unlabelled chemokines were purchased from PeproTech. CXCL10 was labelled with Cy5 using the Amersham QuickStain Protein Labeling Kit (GE Healthcare Life Sciences, Marlborough, MA, USA). Alexa Fluor 647-labelled CCL2 (CCL2-AF647) was purchased from Almac (Craigavon, UK).

### 2.2. Chemokine Processing by Dipeptidyl Peptidase 4

CCL5, CCL2, CXCL10, CXCL11 and CXCL12 chemokines (9 µM) were incubated with recombinant dipeptidyl peptidase 4 (CD26) (200 U) in Tris/HCl 50 mM pH 7.5 + 1 mM EDTA for 1 h at 37 °C in the presence or absence of the sitagliptin (10 µM) (Sigma Aldrich, St. Louis, MO, USA). The efficiency of processing was verified by MALDI-TOF analysis using a RapifleX, Bruker Daltonics instrument (Billerica, MA, USA) in positive ion mode and in reflectron mode.

### 2.3. Chemokine-Induced β-Arrestin Recruitment

Chemokine-induced β-arrestin recruitment to receptors was monitored by NanoLuc complementation assay (NanoBiT) [43,44,45] or by NanoBRET using mNeonGreen as acceptor molecule.

NanoBiT: HEK293T or U87.MG cells were co-transfected with pNBe vectors encoding chemokine receptors C-terminally fused to SmBiT and human β-arrestin-1/2 N-terminally fused to LgBiT. Twenty-four hours after transfection cells were harvested, incubated 25 min at 37 °C with Nano-Glo Live Cell substrate (1:200) and upon addition of chemokines at the indicated concentrations, β-arrestin recruitment was evaluated with a Mithras LB940 luminometer (Berthold Technologies, Bad Wildbad, Germany). Each point corresponds to average values acquired for 20 min, represented as percentage of maximum full agonist response.

NanoBRET: HEK293T cells were co-transfected with pNeonGreen and pNLF vectors encoding ACKR2 C-terminally fused to mNeonGreen and β-arrestin-1 N-terminally fused to Nanoluciferase. Twenty-four hours after transfection cells were harvested and upon simultaneous addition of Nano-Glo Live Cell substrate (1:200) and chemokines, BRET signal was measured with a Mithras LB940 luminometer (Berthold Technologies) using a 460/70 BP filter for Nanoluciferase and a 515/40 BP filter for mNeonGreen signal.

### 2.4. Chemokine Binding

HEK293T and HEK-ACKR2 cells were incubated with CXCL10-Cy5 at indicated concentrations for 45 min at 37 °C, then washed twice with FACS buffer (PBS, 1% BSA, 0.1% NaN_3_). Dead cells were excluded using Zombie Green viability dye (BioLegend). ACKR2-negative HEK293T cells were used to evaluate non-specific binding of CXCL10-Cy5. For binding competition with unlabelled chemokines (50 nM or 10 nM), the signal obtained for CXCL10-Cy5 (100 ng/mL) or CCL2-AF647 (30 ng/mL) in the absence of unlabelled chemokines was used to define 100% binding. Ligand binding was quantified by mean fluorescence intensity on a BD FACS Fortessa cytometer (BD Biosciences, Franklin Lakes, NJ, USA).

### 2.5. Chemokine-Induced Receptor Mobilisation to the Plasma Membrane

Ligand-induced receptor mobilisation to the plasma membrane was monitored by NanoBRET. A total of 5 × 10^6^ HEK293T cells were seeded in 10 cm dishes and co-transfected with plasmids encoding ACKR2 C-terminally tagged with Nanoluciferase and mNeonGreen C-terminally tagged with the plasma membrane targeting polybasic sequence and prenylation signal sequence from K-RAS splice variant b [46]. Twenty-four hours after transfection, cells were distributed into black 96-well plates (1 × 10^5^ cells per well) and treated with chemokines (100 nM). After 45 min incubation at 37 °C, coelenterazine H (10 µM) was added, and donor emission (460 nm) and acceptor emission (535 nm) were immediately measured on a GloMax plate reader (Promega, Madison, WI, USA).

### 2.6. Chemokine-Induced Receptor-Arrestin Delivery to Endosomes

Ligand-induced receptor-arrestin delivery to early endosomes was monitored by NanoBRET. In brief, 5 × 10^6^ HEK293T cells were seeded in 10 cm dishes and co-transfected with plasmids encoding ACKR2, β-arrestin-2 N-terminally tagged with Nanoluciferase and FYVE domain of endofin interacting with phosphatidylinositol 3-phosphate (PI3P) in early endosomes [46,47], N-terminally tagged with mNeonGreen. Twenty-four hours after transfection, cells were distributed into black 96-well plates (1 × 10^5^ cells per well) and treated with full-length or processed chemokines. After 2 h incubation at 37 °C, coelenterazine H (10 µM) was added, and donor emission (460 nm) and acceptor emission (535 nm) were immediately measured on a GloMax plate reader (Promega).

### 2.7. Chemokine Scavenging

Chemokine depletion from the extracellular space was quantified by ELISA. HEK293T and HEK-ACKR2 cells were incubated 8 h at 37 °C with chemokines at 0.3 and 30 nM. Chemokine scavenging by ACKR2 was evaluated by quantifying the concentration of chemokines remaining in the supernatant using commercially available ELISA kits (CXCL10 R&D Systems, CCL5 BioLegend and CXCL11 Peprotech, Rocky Hill, NJ, USA) and was expressed as the percentage of input chemokine concentrations.

### 2.8. Chemokine Internalization

Chemokine internalization using labelled CXCL10 or CCL2 was visualized by imaging flow cytometry as previously described [7]. HEK.293T or HEK-ACKR2 cells were incubated 15 min at 37 °C in the presence or absence of unlabelled chemokines (200 nM) after which Cy5-labelled CXCL10 (100 nM) or AF647-labelled CCL2 (100 ng/mL) was added for 45 min at 37 °C. Cells were washed twice with FACS buffer. Dead cells were excluded using Zombie Green viability dye (BioLegend). Images of 1 × 10^4^ in-focus living single cells were acquired with an ImageStream MKII imaging flow cytometer (Amnis Luminex, Austin, TX, USA) using 60× magnification. Samples were analysed using Ideas6.2 software. The number of spots per cell was determined using a mask-based software wizard.

For confocal microscopy, 4 × 10^4^ HEK-ACKR2 cells/well were seeded on poly-L-lysine coated 8-well chamber slides (µ-Slide 8 well, Ibidi, Fitchburg, WI, USA). After 36 h, cells were incubated 2 h at 37 °C with 100 nM Cy5-labelled chemokines (CXCL10, CXCL11 or CCL2) and co-incubated one additional hour with 750 nM LysoTracker™ Red DND-99 (ThermoFisher, Schwerte, Germany). Cells were then washed twice with PBS, fixed with 3.5 % (*w/v*) paraformaldehyde for 20 min at room temperature and washed again twice with PBS. Nuclear staining was performed with Hoechst 33342 dye (1 µg/mL) for 20 min at room temperature, and cells were washed 3 times with PBS. Images were acquired on a Zeiss LSM880 confocal microscope using a 63× oil-immersion objective and Zen Black 2.3 SP1 software (Zeiss, Jena, Germany). Representative cells from 12 image acquisitions of three independent experiments are shown.

### 2.9. Inhibition of Chemokine Uptake by Anti-mACKR2 Antibodies

HEK-mACKR2 or B16-F10 cells were incubated 45 min at 37 °C with Cy5-labelled mCXCL10 (100 nM) in the presence or absence of the polyclonal goat anti-mACKR2 antibody (50 µg/mL) (ab1656, Abcam) or goat IgG control antibody (ab37373, Abcam) and the secondary donkey anti-goat-AF647 antibody (Jackson ImmunoResearch, West Grove, PA, USA). Dead cells were excluded using Zombie Green viability dye (BioLegend). Ligand uptake was quantified by mean fluorescence intensity on a BD FACS Fortessa cytometer (BD Biosciences). Inhibition of mCXCL10 scavenging by anti-mACKR2 was expressed as the percentage relative to conditions where the antibody was absent.

### 2.10. Data and Statistical Analysis

Concentration–response curves were fitted to the four-parameter Hill equation using an iterative, least-squares method (GraphPad Prism version 8.0.1) to provide EC_50_ values and standard errors of the mean. All curves were fitted to data points generated from the mean of at least three independent experiments. All statistical tests, i.e., *t*-tests, ordinary one-way ANOVA and post hoc analysis, were performed with GraphPad Prism 8.0.1. *p*-values are indicated as follows: * *p* < 0.05, ** *p* < 0.01, *** *p* < 0.001, **** *p* < 0.0001.

## 3. Results and Discussion

The pairing of ACKR2 with CC chemokines dates back to when many chemokines, especially the CXC chemokines, had not yet been known or available [12,15,48]. Recent identification of CCL20 and CCL22 as ligands for ACKR4 [49,50] demonstrates that some pairings within the complex chemokine–receptor interaction network may have been overlooked. Several reports point to increased CXC chemokine levels in ACKR2-deficient mice [51,52], and an indirect crosstalk between the orphan CXCL14 and ACKR2 has recently been described [53]. These observations prompted us to re-evaluate the ability of ACKR2 to scavenge chemokines also from the CXC family.

**Figure 1:**
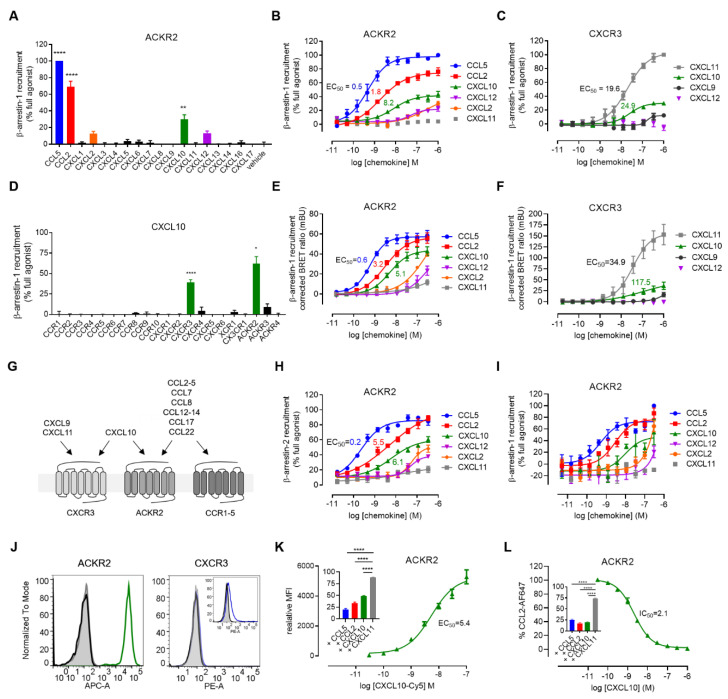
ACKR2 activation by CXCL10. (**A**) β-arrestin-1 recruitment to ACKR2 in response to all known human CXC chemokines (100 nM) monitored by NanoBiT-based assay. CCL2 and CCL5 were used as positive control chemokines. (**B**) β-arrestin-1 recruitment to ACKR2 by the CXC chemokines CXCL2, CXCL10 and CXCL12 monitored by NanoBiT, showing the concentration–response relationship. CXCL11 was used as negative control. (**C**) β-arrestin-1 recruitment to CXCR3 induced by its cognate ligands CXCL9, CXCL10 and CXCL11 monitored by NanoBiT. CXCL12 was used as negative control. (**D**) β-arrestin-1 recruitment to all known chemokine receptors in response to CXCL10 (100 nM). (**E**,**F**) β-arrestin-1 recruitment to ACKR2 (**E**) and CXCR3 (**F**) monitored by NanoBRET. (**G**) Schematic representation of chemokine–receptor interactions between ACKR2, CXCR3 and the CC receptors CCR1, CCR2, CCR3, CCR4 and CCR5, including the newly identified pairing between CXCL10 and ACKR2. (**H**) β-arrestin-2 recruitment to ACKR2 by the CXC chemokines CXCL2, CXCL10 and CXCL12 monitored by NanoBiT. (**I**) β-arrestin-1 recruitment to ACKR2 by the CXC chemokines CXCL2, CXCL10 and CXCL12 monitored by NanoBiT in U87.MG cells. (**J**) Flow cytometry analysis of cells used in the binding studies, left panel: ACKR2 surface expression in HEK-ACKR2 (green histogram) and the parental HEK293T cell line (grey- filled histogram) evaluated using the ACKR2-specific mAb (clone 196124) or the corresponding isotype control (black histogram); right panel: CXCR3 surface expression in HEK-ACKR2 evaluated using the CXCR3-specific mAb (clone 1C6) (blue histogram) and the corresponding isotype control (black histogram). Unstained cells are represented as grey filled histogram. (inset) Positive control surface expression staining for CXCR3 in HEK293T cells transiently transfected with a CXCR3-encoding vector, using CXCR3-specific mAb (clone 1C6) (blue histogram) and the corresponding isotype control (black histogram). (**K**) Binding of Cy5-labelled CXCL10 to HEK-ACKR2 cells. (inset) Binding competition (100 ng/mL CXCL10-Cy5) with unlabelled chemokines (50 nM). (**L**) Binding competition of unlabelled CXCL10 with Alexa Fluor 647-labelled CCL2 (30 ng/mL) on HEK-ACKR2 cells. (inset) Binding competition with unlabelled chemokines (10 nM). EC_50_ and IC_50_ values for concentration–response curves (**B**–**L**) are indicated (nM). All NanoBiT and NanoBRET assays were conducted in HEK293T cells except for (**I**) for which U87.MG cells were used. Data points represent mean ± SEM of three independent experiments. * *p* < 0.05, ** *p* < 0.01, **** *p* < 0.0001 by one-way ANOVA with Dunnett (**A**,**D**) and Bonferroni (**K**,**L**) post hoc tests.

First, we assessed the activity of the 16 human CXC chemokines (100 nM) towards ACKR2 by monitoring their ability to induce β-arrestin-1 recruitment using Nanoluciferase complementation-based assay (NanoBiT). Our screening revealed that at least three CXC chemokines, namely CXCL2, CXCL10 and CXCL12, are capable of inducing β-arrestin-1 recruitment to ACKR2. However, only CXCL10 reached statistical significance in this assay (Figure 1A).

To evaluate the functional relevance of the interactions between these chemokines and ACKR2, especially in light of a possible scavenging function, we next performed an in-depth analysis of intracellular events and monitored the fate of the chemokines and receptor following their interactions.

CXCL2 and CXCL12 consistently showed reduced potency and efficacy in β-arrestin recruitment towards ACKR2 compared to CXCL10 or to the activity they display towards their already known receptors [45,54,55,56] (Figure 1B,E,H,I). Given this limited activity, they were not further investigated. CXCL10, however, showed a strong potency towards ACKR2 (EC_50_ = 8.2 nM, pEC_50_ = 8.08 ± 0.14) and induced approximately half of the maximal response compared to the full agonist CCL5 (Figure 1B). This partial agonist behaviour of CXCL10 was reminiscent of the activity towards its long-established signalling receptor CXCR3 relative to the full agonist CXCL11 (Figure 1C,F,G) [57,58]. The potency of CXCL10 towards ACKR2 appears approximately 3 times stronger than towards CXCR3 (EC_50_= 24.9 nM, pEC_50_ = 7.60 ± 0.12), consistent with a potential scavenging role of ACKR2. In NanoBRET, the potency of CXCL10 towards ACKR2 (EC_50_ = 5.1 nM, pEC_50_ = 8.29 ± 0.11) was close to that of CCL2 and approximately 20-fold stronger than towards CXCR3. The efficacy of CXCL10 in this assay reached approximately 70% of the maximal signal measured with CCL5 (Figure 1E,F). Similar observations were made for the recruitment of β-arrestin-2 (Figure 1H) and were further confirmed in a different cellular background (Figure 1I). Moreover, the screening of CXCL10 on 23 chemokine receptors showed that CXCR3 and ACKR2 are the only human receptors activated by CXCL10 (Figure 1D). Fluorescently labelled CXCL10 also strongly and specifically bound to HEK293T cells expressing ACKR2 (IC_50_ = 5.4, pIC_50_ = 8.27 ± 0.09) (Figure 1J,K) and was only displaced by ACKR2-related chemokines CCL5, CCL2 and by CXCL10 itself (Figure 1K inset). Inversely, binding competition studies showed that CXCL10 was able to fully displace fluorescently labelled CCL2 from the receptor with an IC_50_ of 2.1 nM (pIC_50_ = 8.68 ± 0.03) (Figure 1L).

**Figure 2:**
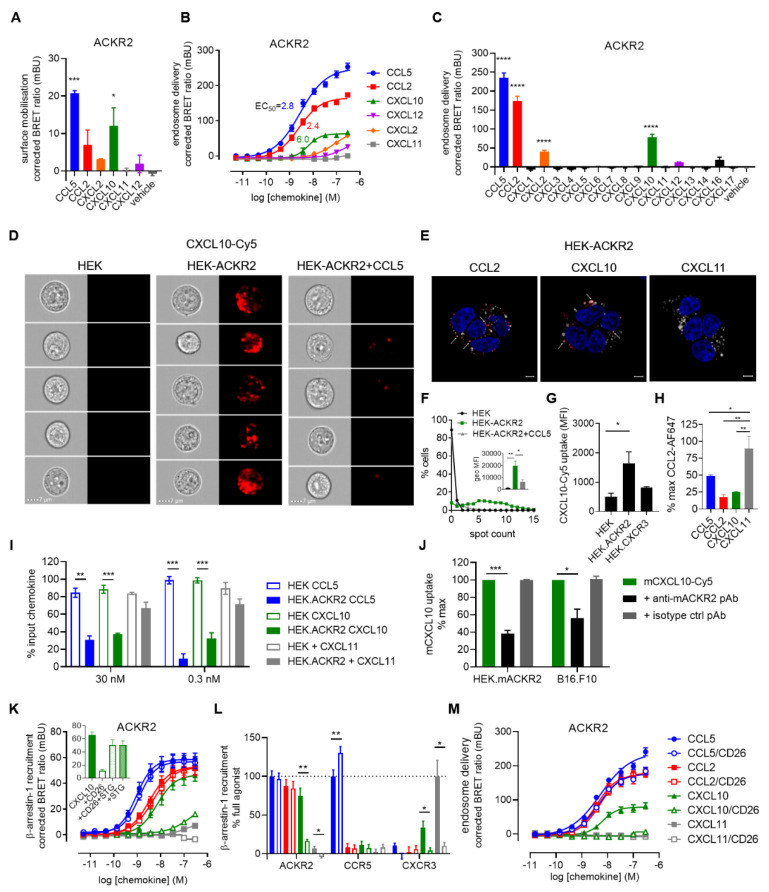
CXCL10 scavenging by ACKR2. (**A**) ACKR2 mobilisation to the plasma membrane in response to chemokines (100 nM) monitored by NanoBRET-based assay. (**B**,**C**) β-arrestin-1/ACKR2 complex delivery to the early endosomes in response to the CXC chemokines CXCL2, CXCL10 and CXCL12 (B) or the 16 human CXC chemokines (100 nM) (**C**) monitored by NanoBRET-based assay. CCL2 and CCL5 were used as positive control chemokines. (**D**–**F**) Uptake of fluorescently labelled CXCL10 by ACKR2-expressing cells visualized by imaging flow cytometry (**D**,**F**) and confocal microscopy (**E**). (**D**) HEK, HEK-ACKR2 or HEK-ACKR2 cells pre-treated with CCL5 at saturating concentration (200 nM) were stimulated for 45 min at 37 °C with 100 nM (Cy5)-labelled CXCL10 (CXCL10-Cy5, red channel). Five representative cells for each condition are shown (10,000 events recorded). Scale bar: 7 µm. (**F**) Percentage of cells from (**D**) with a given number of distinguishable vesicle-like structures (spots), as well as the geometrical mean fluorescence intensity (MFI) for the red channel were determined (inset). Data shown are representative of three independent experiments and for inset, mean ± SEM of three independent experiments. (**E**) Cellular localization of Cy5-labelled chemokine (red) following HEK-ACKR2 stimulation (100 nM) for 2 h monitored by fluorescent confocal microscopy. Lysosomes and nucleic DNA were stained using LysoTracker™ Red DND-99 (white) and Hoechst 33342 (blue), respectively. Pictures are representative of 12 acquired images from three independent experiments. Scale bar: 5 µm. Arrows highlight colocalization of Lysotracker and chemokine-Cy5 signal. (**G**) Uptake of Cy5-labelled chemokine (100 nM) by HEK cells transfected or not with equal amounts of ACKR2 or CXCR3 vectors analysed by imaging flow cytometry as described in (**D**). (**H**) Binding competition between Alexa Fluor 647-labelled CCL2 (100 ng/mL) and unlabelled chemokines (100 nM) in HEK-ACKR2 analysed by imaging flow cytometry. (**I**) ACKR2-mediated depletion of extracellular CXCL10 monitored by ELISA. Chemokines in the supernatant of HEK293T cells expressing or not ACKR2 were quantified after 8 h stimulation, and expressed as percentage of the input concentrations (30 nM and 0.3 nM). CCL5 and CXCL11 were used as positive and negative controls, respectively. Data points represent mean ± SEM of three independent experiments. (**J**) Inhibition of mACKR2-mediated mCXCL10 uptake by neutralizing antibodies. Cy5-labelled mouse CXCL10 (mCXCL10-Cy5) (100 nM) was incubated with HEK-mACKR2 or B16.F10 in the presence of mACKR2-specific polyclonal antibody (Ab1656) or corresponding isotype control (Ab37373) for 45 min at 37 °C and analysed by flow cytometry. (**K**–**M**) Impact of chemokine N-terminal processing by dipeptidyl peptidase 4 (DPP4/CD26) on the activation of ACKR2 and related receptors CXCR3 and CCR5 and ACKR2 delivery to the endosomes. (**K**,**L**) β-arrestin-1 recruitment to ACKR2 by processed chemokines monitored by NanoBRET. (**L**) Comparison of the impact of N-terminal processing on the ability of CXC and CC chemokines (100 nM) to induce β-arrestin-1 recruitment to ACKR2, CXCR3 and CCR5. (Inset) Comparison of ACKR2 activity induced by unprocessed CXCL10 or CXCL10 treated with CD26 in the presence or absence of its specific inhibitor, sitagliptin (STG) (10 µM) or with STG alone, demonstrating no interference between CD26 and the ACKR2-CXCL10 interaction. (**M**) β-arrestin-1/ACKR2 complex delivery to the early endosomes in response to processed chemokines monitored by NanoBRET. * *p* < 0.05, ** *p* < 0.01, *** *p* < 0.001, **** *p* < 0.0001 by one-way ANOVA with Dunnet (**A**,**C**) and Bonferroni (**H**) post hoc tests or repeated measures one-way ANOVA with Bonferroni post hoc test (**J**) and two-tailed unpaired Student’s *t*-test (**I**).

The ability of ACKR2 to mediate CXCL10 scavenging and control its extracellular concentration was then analysed. CXCL10 stimulation resulted in rapid mobilisation of intracellular ACKR2 to the plasma membrane reminiscent of the activity of CC chemokines [59,60] (Figure 2A). The CXCL10-induced receptor mobilisation was followed by its delivery to the endosomes with an EC_50_ of 6.0 nM (pEC_50_ = 8.22 ± 0.06) (Figure 2B,C). Imaging flow cytometry also revealed specific and efficient uptake of labelled CXCL10 by ACKR2-expressing cells. A notably higher number of distinguishable intracellular vesicle-like structures and mean fluorescent intensity were observed compared to HEK293T cells or HEK-ACKR2 cells pre-treated with CCL5 (Figure 2D,F). Confocal microscopy further confirmed CXCL10 uptake and in addition showed its distribution within acidic intracellular vesicles (Figure 2E). Moreover, the uptake of CXCL10 by ACKR2 was more efficient compared to that by CXCR3, consistent with the stronger potency of CXCL10 towards ACKR2 and the possible scavenging function (Figure 2G). As an additional selectivity control, CXCL10—just like CCL5 and CCL2—was able to compete with the uptake of fluorescently labelled CCL2 by ACKR2-expressing cells in imaging flow cytometry (Figure 2H). Importantly, the ACKR2-driven intracellular accumulation of CXCL10 was also associated with a reduction of its availability in the extracellular space as demonstrated by ELISA quantification. The efficiency of ACKR2-driven CXCL10 scavenging was similar at high (30 nM) and low (0.3 nM) chemokine concentrations (Figure 2I) and was comparable to the depletion of CCL5, while no reduction was observed for CXCL11. The interaction between CXCL10 and ACKR2 was also observed with the murine counterparts, as illustrated by the uptake of labelled murine CXCL10 (mCXCL10) by HEK-mACKR2 cells or the mouse melanoma cell line B16.F10, which was partially inhibited by mACKR2-specific polyclonal antibody but not the isotype control (Figure 2J).

Similar to many other CC and CXC chemokines, CXCL10 was shown to be subject to post-translational modification by proteolytic enzymes [61]. In particular, N-terminal cleavage by the dipeptidyl peptidase 4 (DPP4 or CD26) was demonstrated to turn CXCL10 from CXCR3 agonist to antagonist [62]. Based on recent reports demonstrating that, in contrast to CXCR3, ACKR3 is responsive to DPP4-inactivated CXCL11 [45], the impact of the CXCL10 N-terminal processing on ACKR2 activation was evaluated and compared to CXCR3. We observed that, in contrast to CC chemokines, truncation of CXCL10 drastically reduced its ability to induce β-arrestin-1 recruitment to ACKR2 (Figure 2K,L) and subsequent receptor targeting to the early endosomes (Figure 2M), indicating that CXCL10 N-terminal residues are critical for its activity towards ACKR2 [60,63]. The uptake of CD26-processed CXCL10 by ACKR2-positive cells was also highly reduced and, similar to the full-length chemokine, competed out by non-truncated CXCL10 or ACKR2-related CC chemokines (data not shown). These results, in addition to partial agonist behaviour of CXCL10, point to distinct ACKR2 interaction and activation modes compared to CC chemokines. This may be attributed to notable differences in the N terminus orientation and occupation of the receptor binding pockets of CXC and CC chemokines [64].

## 4. Conclusions

In conclusion, our study shows that CXCL10 is a novel ACKR2 ligand. CXCL10 is one of the most important inflammatory CXC chemokines and is involved in many physiological and pathological processes such as angiogenesis, chronic inflammation, immune dysfunction, tumour development and dissemination [65,66], in which ACKR2 has also been shown to play critical roles [35]. Together with CCL5, CXCL10 is a key player in driving NK cells and CD8+ T cells into the tumour bed [37,38,40,41]. This novel pairing consequently adds an unforeseen level of complexity to ACKR2 functions and a new level of CXCL10 regulation and could thus encourage re-examination of previous studies taking into account CXCL10–ACKR2 interactions (Figure 1G) [27,51,52,65,67].

The ability to bind and respond to both CXC and CC chemokines has already been reported for ACKR1 [68], ACKR3 [69] and ACKR4 [70], although this property has recently been challenged for the latter. Here, we identified an agonist CXC ligand for ACKR2, which until now has been recognised for binding inflammatory CC chemokines only. Therefore, such cross-family spectrum of chemokine ligands, uncommon among the classical chemokine receptors, seems to represent an additional functional property of ACKRs [2] besides their inability to trigger G protein signalling. Overall, this study highlights that a systematic reassessment of chemokine–receptor pairings for both long-established and recently deorphanized receptors may be necessary, as important interactions may have been overlooked.

## Data Availability

All data are available from the corresponding author upon reasonable request.
